# Measuring the quality of life of family carers of people with dementia: development and validation of C-DEMQOL

**DOI:** 10.1007/s11136-019-02186-w

**Published:** 2019-04-27

**Authors:** Anna Brown, Thomas E. Page, Stephanie Daley, Nicolas Farina, Thurstine Basset, Gill Livingston, Jessica Budgett, Laura Gallaher, Yvonne Feeney, Joanna Murray, Ann Bowling, Martin Knapp, Sube Banerjee

**Affiliations:** 1 School of Psychology, University of Kent, Canterbury, CT2 7NP UK; 2 Centre for Dementia Studies, Brighton and Sussex Medical School, University of Sussex, Brighton, BN1 9RY UK; 3 Lived Experience Advisory Panel, Sussex Partnership NHS Foundation Trust, Hove, BN3 7HQ UK; 4 Division of Psychiatry, University College London, London, W1T 7NF UK; 5 Institute of Psychiatry, Psychology & Neuroscience, King’s College London, London, SE5 8AF UK; 6 Health Sciences, University of Southampton, Southampton, SO17 1BJ UK; 7 Department of Social Policy, London School of Economics, London, CT1 3LX UK

**Keywords:** Quality of life, Family carer, Dementia, Alzheimer’s disease, Caregiver, Bifactor model, Factor analysis

## Abstract

**Purpose:**

We aimed to address gaps identified in the evidence base and instruments available to measure the quality of life (QOL) of family carers of people with dementia, and develop a new brief, reliable, condition-specific instrument.

**Methods:**

We generated measurable domains and indicators of carer QOL from systematic literature reviews and qualitative interviews with 32 family carers and 9 support staff, and two focus groups with 6 carers and 5 staff. Statements with five tailored response options, presenting variation on the QOL continuum, were piloted (*n* = 25), pre-tested (*n* = 122) and field-tested (*n* = 300) in individual interviews with family carers from North London and Sussex. The best 30 questions formed the C-DEMQOL questionnaire, which was evaluated for usability, face and construct validity, reliability and convergent/discriminant validity using a range of validation measures.

**Results:**

C-DEMQOL was received positively by the carers. Factor analysis confirmed that C-DEMQOL sum scores are reliable in measuring overall QOL (*ω* = 0.97) and its five subdomains: ‘meeting personal needs’ (*ω* = 0.95); ‘carer wellbeing’ (*ω* = 0.91); ‘carer-patient relationship’ (*ω* = 0.82); ‘confidence in the future’ (*ω* = 0.90) and ‘feeling supported’ (*ω* = 0.85). The overall QOL and domain scores show the expected pattern of convergent and discriminant relationships with established measures of carer mental health, activities and dementia severity and symptoms.

**Conclusions:**

The robust psychometric properties support the use of C-DEMQOL in evaluation of overall and domain-specific carer QOL; replications in independent samples and studies of responsiveness would be of value.

**Electronic supplementary material:**

The online version of this article (10.1007/s11136-019-02186-w) contains supplementary material, which is available to authorized users.

## Introduction

Dementia is a syndrome with multiple causes that results in progressive decline in cognitive, social and physical function, with impairment in the skills needed to carry out daily activities. It not only has a devastating impact on those with the illness, but also profoundly changes lives of the family members who provide the majority of daily care, as well as practical and emotional support. An estimated 700,000 family carers^1^ in the UK and 1,590,000 in the USA provide unpaid dementia care that amounts to £11.6 billion and $230.1 billion, respectively, every year, in lost income and out-of-pocket payments [1, 25]. The dementia care system relies on work carried out by family carers, with them being recognized as “the most valuable resource” for people with dementia [8].

While there are positive experiences that can be derived from the caring role in dementia, caring for a family member with dementia often comes at a cost to the carer’s own quality of life (QOL). Anxiety, stress, burden and exhaustion, as well as problems with their own health are common among family carers [17]. Supporting family carers is vital since their care is associated with the prevention of costly interventions that are associated with lower QOL for people with dementia such as hospital admissions [16] and transitions into care homes [2]. Maintaining carers’ mental, physical and economic wellbeing is therefore important to ensure the sustainability of this important resource; provision of support to carers is a key element of national and international dementia strategies [8, 37].

Devising systems, services or interventions intended to support family carers needs to be accompanied by the evaluation of their effectiveness. Quality of life measurement allows the broad evaluation of overall effects of intervention; however, measuring carer QOL robustly in dementia is difficult without a comprehensive and reliable measure suitable for a variety of caring situations and dementia severity. The QOL of family carers of people with dementia is often measured using generic instruments, or by considering psychopathology, burden and the challenges and rewards of caring. Recent systematic reviews [17, 24] have suggested that significant positive and negative impacts may be missed by the use of such approaches. Generic QOL instruments are widely available but lack ‘social relevance’ [17] as they do not refer to issues of importance in dementia care. The only two dementia-specific measures identified in the systematic review of instruments [24] were judged to be either too broad or too nuanced to be used in general settings, as well as having other limitations. The Alzheimer’s Carers’ Quality of Life Instrument (ACQLI) [10], a 30-item checklist of symptoms, conceptualizes QOL as a unidimensional construct, and does not support a more nuanced evaluation of issues. For instance, it cannot be determined to what extent an overall poor QOL score is driven by burden, anxiety or poor relationships with the person with dementia, despite inclusion of these issues at the item level. Moreover, the checklist contains negative issues only, thus omitting the assessment of positive aspects of caring. The Caregiver Quality of Life (CGQOL) instrument [34], an extended 80-item inventory, assesses 10 domains of carer experiences, including the objective characteristics of the caregiving situation (for example, whether the carer assisted with specific activities) in addition to the more direct indicators of QOL such as worry. The comprehensive coverage of the issues is achieved at a cost—the time it takes to complete the CGQOL is significant, which limits its use. The evidence of the instrument’s validity is also limited—factor-analytic examination has only been conducted at the subscale level, lacking confirmation of dimensionality at the item level. Both reviews concluded that there was a need to develop a new measure of QOL specific to caring for a family member with dementia.

In this project, we set out to develop such a measure from the ‘bottom-up’, involving the population of interest from the outset. In line with the definition of QOL used by the World Health Organization, we focused on the evaluation by the carers of their position in life, assessed *subjectively* in the context of their culture, values, goals, expectations, standards and concerns, not *objective* demands placed on them. Drawing on literature about areas of impact on carer’s QOL [11, 17], we paid attention to positive as well as negative issues. Carers can experience personal fulfilment, an improved sense of self-worth and greater closeness with the person with dementia, so enhancing their QOL [22]. However, they may also find challenges in looking after someone with dementia. The development was designed to ensure comprehensive coverage of relevant domains, while prioritizing psychometric efficiency and usability in research practice.

## Aims

We aimed to develop a condition-specific measure of the QOL of family carers of people with dementia, applicable across the range of caring situations and severity in dementia. The measure had to provide: (i) comprehensive coverage of issues relevant to the QOL of family carers of people with dementia; (ii) accurate measurement of overall QOL and subdomains; and (iii) usability and efficiency, limiting the cognitive, emotional and time burdens on carers.

## Methods

### Stage 1: Identifying factors relevant to family carer’s QOL

First we identified aspects of good and poor QOL of family carers of people with dementia to form the basis of measurement using qualitative research [7] and a systematic review of the literature [11]. Qualitatively, we generated a conceptual framework through an open exploration of positive and negative issues affecting QOL in individual interviews with 32 family carers and nine clinical staff who work in dementia services and in two focus groups with six family carers and five staff. To capture the breadth of issues, our definition of family carer at Stage 1 was intentionally inclusive of married and unmarried partners, children, siblings, extended family and close friends who were currently providing at least 4 h of care each week for someone with dementia. Interviews with carers explored their experience of and feelings towards caregiving, and perceived impact of these on their QOL. To generate a rounded and comprehensive view of the factors affecting the QOL of family carers we also included interviews with experienced professionals—nurses, occupational therapists, a psychologist and voluntary sector staff. Our discussions with them focused on their experiences of working with family carers and their appraisal of carer QOL, not that of paid carers or of other staff. From the interviews and focus groups, we identified 12 themes presented in Table 1 and grouped them according to focus on either: *Person with dementia* (themes 1–3), *Carer* (themes 4–8) or *External environment* (themes 9–12).

**Table 1:** Conceptual map of qualitative themes, factors associated with carer QOL and constructs measured in an existing condition-specific measure (CGQOL) and in C-DEMQOL (initial and empirically confirmed)

Factors associated with carer QOL [11]	Constructs measured in CGQOL [34]	12 themes from qualitative interviews	C-DEMQOL 7 working constructs	C-DEMQOL 5 final constructs
Carer-patient relationship		1. Relationship with the person with dementia	Relationship with the person with dementia	Carer-patient relationship
Carer self-efficacy	Benefits of caregiving	5. Acceptance of the caring role	Carer role (appraisal of own role, self-efficacy, acceptance)	
	Spirituality and faith	6. Finding meaning		
Dementia characteristics	Caregiver feelings	2. [feelings about] Change in the person with dementia	Carer health (physical and emotional)	Carer wellbeing
Carer health; Carer emotional wellbeing		7. Carer health (physical and emotional)		
Demands of caring	Assistance in IADLS/ADLS^(a)^; Demands of caregiving	3. Demands of caregiving	Carer responsibilities (demands of caregiving, burden)	Meeting personal needs
	Role limitations due to caregiving	12. Role conflict		
Carer independence	Personal time	4. Personal freedom	Carer personal needs (need for personal time and space)	
Future	Worry	8. Expectations of the future	Feelings about future	Confidence in the future
Support received	Family interaction	9. Evaluation of support	Carer support (from family, professionals and community)	Feeling supported
		10. Weight of responsibility		
		11. Family and social networks		

^a^Objective demands rather than subjective assessments of the impact of demands

To cross-validate the qualitative findings, we mapped the 12 themes against factors found to influence carer QOL in our systematic review of literature [11], and against the scales included in the most comprehensive measure to date, CGQOL [34], identified in our systematic review of instruments measuring carer QOL [24]. No new themes emerged in the mapping exercise; and the resulting thematic map (Table 1) was adopted to generate an initial item pool.

### Stage 2: Generation and piloting of items

We extracted quotes from family carer interviews indicating positive and negative QOL under the 12 themes of our conceptual framework, and developed them into behavioural indicators. For example, within the theme Personal Freedom, ‘being rarely able or completely unable to undertake preferred activities’ indicated low QOL and ‘being able to undertake preferred activities on a regular basis’ indicated high QOL. The indicators served as the main content source for questionnaire items, which were generated in an iterative process including workshops with the authors and reference to a study-specific Lived Experience Advisory Panel (LEAP) of family carers.

#### Response format

We specified that items should be statements expressed in the first person, with response options presenting variation of the feeling/behaviour expressed on the QOL continuum, from best QOL to worst. This gives five-point ordinal response scales tailored to the items. For example, the below question was created to measure the extent of carer personal freedom, based on the behavioural indicators identified earlier:

Thinking of my ability to do things I enjoy, I have felt …

- free to do them when I want
- restricted in a little way by my caring duties
- restricted to some extent by my caring duties
- restricted a lot by my caring duties
- unable to do them due to my caring duties

This ‘expanded’ response format [39] has several advantages. First, as the above example illustrates, more content can be covered by including several behavioural indicators under the same stem. To present the same content in an ‘agree-disagree’ or any other fixed format, several items are needed. Second, inclusion of positive and negative graded states eliminates the need for negatively worded item stems, which often cause confusion and have been shown to produce unwanted method effects such as distortions to factor structures [18]. Third, it has been shown that by making participants pay more attention to the item content, the expanded formats reduce carelessness in responding, improving validity [39].

### Developing item pool, establishing measured constructs and piloting

Theme by theme, an initial pool of 77 items was generated. At the item generation stage, it became clear that some items written for different conceptual themes (with seemingly distinct semantics) yielded similar behavioural manifestations and hence indicated the same construct. The item generation process thus informed a conceptual review of the 12 themes to establish QOL *constructs* to be measured by the future questionnaire. After the content review, the items were organized under seven working constructs. Table 1 lists the working constructs and shows how they map to the 12 themes and constructs identified in the systematic reviews.

The item pool was piloted with 25 family carers in one-to-one interviews, and reviewed by the LEAP. Detailed feedback was collected on appropriateness and applicability of item wording, tailored response format and response options, presentation, face validity, missing issues and time taken to complete. The feedback was collated and qualitatively analysed; and amendments were made, resulting in a 52-item preliminary questionnaire version.

### Stage 3: Pre-trial and the development of field version

#### Participants and procedure

From this stage on, we recruited only primary carers, that is, people who had primary responsibility for someone with dementia. To broaden the demographics of our sample, we circulated study information across clinical and voluntary services in two target areas—Sussex (mainly rural or small-town population, predominantly white) and North London (urban population, with more mixed ethnic backgrounds). One-hundred-and-twenty-two family carers of people with a clinical diagnosis of dementia were recruited from Sussex (*n* = 72) and North London (*n* = 50). There were 69 co-resident and 53 non-resident carers, including those responsible for relatives with dementia in care homes. The family carers were visited in their own homes, where researcher-administered interviews took place. The interviews started with the C-DEMQOL 52-item preliminary version, followed by a battery of sociodemographic and validation measures presented in Appendix, which were later used in the field trial (Stage 4).

#### Analyses

Participant feedback and responses were analysed using the protocol for evaluating the field version (see Stage 4—Analyses) except, given the C-DEMQOL factorial structure had yet to be established, Exploratory Factor Analysis (EFA) with ordinal variables was conducted. Parallel Analysis based on the polychoric correlations of item responses [33] favoured a five-factor solution. Obliquely rotated five factors were readily interpretable, with three factors directly reflecting the working constructs *carer health*, *feelings about future* and *carer support*, and two further factors presenting amalgamations of the remaining four working constructs. Specifically, the working constructs *carer responsibilities* and *carer personal needs* were empirically indistinguishable in this model; the same was the case for *carer*-*patient relationship* and *carer role*. The five domains of QOL identified at this stage were adopted for all further development; their relationship to the original themes and working constructs is presented in Table 1.

Strong positive correlations between the domains warranted fitting an exploratory bifactor model, with one general and five orthogonal specific factors, to facilitate interpretation of common variance in all items as due to overall QOL, and remaining common variance due to specific domains of QOL. For the C-DEMQOL field version, items were selected with positive participant feedback and salient factor loadings on the general QOL factor and one domain-specific factor. The reduced version was checked for reliability using omega coefficients (for detail, see Stage 4—Analyses), and for convergent/discriminant validity using the validation measures. Forty items were retained with at least seven to measure each QOL domain.

### Stage 4: Field trial and psychometric evaluation of the field version

#### Participants and procedure

Three hundred family carers of people with a clinical diagnosis of dementia, all new to the study, were recruited from Sussex (*n* = 162) and North London (*n* = 138). These again were primary carers, from a variety of backgrounds and caring situations summarized in Table 2. The carers were visited in their own homes, where researcher-administered interviews took place.

**Table 2:** Sociodemographic characteristics of the field-test carer sample, *n* = 300

Characteristic	Valid *N*	Categories	*N*
Carer sex	299	Female	218
		Male	81
Carer age	298	Min	21
		Max	90
		Median	62
Carer ethnicity	298	White British	253
		White Other	26
		Black/African/Caribbean	9
		Mixed Ethnic Background	5
		Indian/Bangladeshi	2
		Arab	1
		Other	2
Carer employment status*	299	Paid employment	109
		Retired	100
		Full-time carer	65
		Housewife/husband	11
		Volunteer	9
		Unemployed	5
Carer relationship to the person with dementia	299	Son/daughter	148
		Spouse/long term partner	128
		Family friend	4
		Sibling	3
		Other family member	2
		Other	14
Co-residence with person with dementia	299	Co-resident	151
		Non-resident	148
Type of dementia	274	Alzheimer’s disease	159
		Mixed	52
		Vascular dementia	36
		Frontotemporal dementia	10
		Lewy body dementia	6
		Other	11

*Categories of Carer Employment Status reflect carers’ view of their role in relation to employment. For example, a spouse carer who has not been in employment during most of their adult life may choose to identify as a housewife/husband; while a spouse who has given up work to provide care may identify as a ‘full-time carer’

#### Measures

The interview started with the 40-item field version of C-DEMQOL. The 40 items were grouped into five sections according to the measured domain and titled: (i) responsibilities and personal needs; (ii) wellbeing; (iii) carer role and relationships with the person with dementia; (iv) feelings about future and (v) carer support. Section-specific written instructions were provided to aid evaluation relevant to certain contexts. The C-DEMQOL questionnaire took between 6 and 60 min to complete, with 50% completing in 15 min or less, and 75% in 20 min or less (mode = 14, mean = 17, SD = 7.3).

The C-DEMQOL administration was followed by the battery of sociodemographic and validation measures (also used earlier in Stage 3). In the list of validation measures presented in Appendix, measures 1–3 focus on the person with dementia (reported by the carer), measures 5–11 focus on the carer (self-reported) and measure 4 (BADLS) is mixed in that each area is assessed for how dependent the person with dementia is (*Dependence*) as well as for how much help the carer provides (*Help*). This is important for further analysis and discussion of external validities.

#### Analyses

Participant feedback was qualitatively analysed to verify the content and face validity. Items that raised questions or concern were highlighted for further examination in conjunction with the following quantitative analyses.

1. *Item descriptive statistics* Items with high missing responses, restricted range, high/low median or low variance were noted.
2. *Factorial structure* was evaluated based on polychoric correlations of ordinal item responses (Graded Response Model [30]). A bifactor structure for C-DEMQOL was assumed by design, and a confirmatory bifactor model was fitted in Mplus 8 [23], using the diagonally-weighted least square estimator with robust standard errors. Model fit was evaluated using the *χ*^2^ statistic (with non-significant p-values indicating exact fit), Root Mean Square Error of Approximation (RMSEA, with values 0.06 or smaller indicating close fit), Comparative Fit Index (CFI, with values 0.95 or greater indicating close fit) and Square Root Mean Residual (SRMR, with values 0.08 or smaller indicating close fit) [14]. Items with low loadings on the general QOL factor or cross-loadings outside of the hypothesized domain-specific factor were noted.
3. *Item information functions* were assessed for contributions to measurement of the general and specific factors [19]. Items contributing little information or informative only in a limited trait range were noted.

Items that performed poorly in participant feedback or the above statistical criteria were shortlisted for removal. The shortlist underwent thorough content review, and consultation with the LEAP, to ensure that the remaining items provided sufficient domain coverage. This generated a final 30-item version of C-DEMQOL.

### Stage 5: Confirmation and psychometric evaluation of C-DEMQOL

The psychometric properties of the final 30-item version of C-DEMQOL were established based on the bifactor model as follows.

1. *Reliability* of C-DEMQOL sum scores (total and subdomain) were assessed using the model-based coefficient *omega* [28]. Attribution of score variance due to general or domain-specific factor was evaluated using *omega hierarchical* (omegaH or *ω*_h_) and *omega hierarchical subscale* (omegaHS or *ω*_hs_) as appropriate [28].
2. *Appropriateness of reporting subdomain scores* (in addition to the total score) was appraised using the proportional reduction in mean squared error based on total scores (PRMSE_TOT_) [26]. If PRMSE_TOT_ are smaller than the respective subscale reliabilities (assessed by *omega*), subscale scores add information over the general QOL, and can be reported.
3. *Standard Errors of Measurement* (SE_M_) for each scored construct were computed from respective omega coefficients and scale standard deviations. SE_M_ are more useful in practice than reliability coefficients as they are not population-dependent [19], and are direct measures of the error margin around observed scores.
4. *Construct reliability/replicability* was assessed as the proportion of variability in general and subdomain factors explainable by respective indicator variables, using the index *H* [28], with values around 0.7 and above indicating the latent factor is reliably represented by its indicators. Reliable factors can be used with confidence in latent variable models.
5. *Convergent/discriminant validity* of C-DEMQOL sum scores (total and subdomain) were assessed from observed correlations with the validation measures, using the standard multi-trait multi-method framework [5]. Convergent validity was summarized as the average correlation with external scales measuring conceptually similar constructs, and discriminant validity was summarized as the average correlation with conceptually dissimilar constructs.

## Results: Psychometric properties of C-DEMQOL

### The C-DEMQOL measurement model

**Fig. 1:**
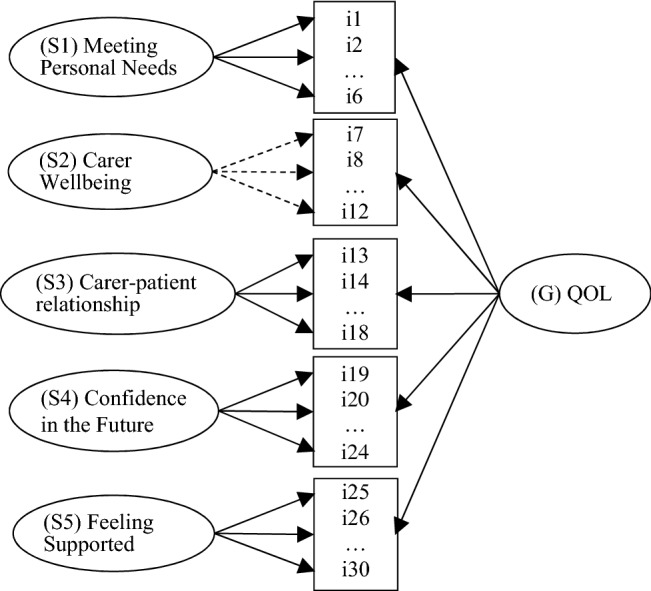
Bifactor measurement model for C-DEMQOL. Dashed arrows signify near-zero empirical factor loadings for specific factor S2 relating to domain Carer Wellbeing (see explanation in text)

A confirmatory bifactor model with one general and five orthogonal specific factors (one per each hypothesized QOL domain) fitted the data reasonably closely according to RMSEA = 0.066, CFI = 0.968 and SRMR = 0.072, although *χ*^2^ = 863.6 (df = 375; *p* < 0.01) was significant. This model illustrated in Fig. 1 yielded near-zero loadings for most items indicating *carer wellbeing*, and fitted no notably better than the more parsimonious model without this domain-specific factor; RMSEA = 0.067, CFI = 0.966 and SRMR = 0.072 (*χ*^2^ = 893.1, df = 381, *p* < 0.01). This suggested that the general QOL factor was sufficient to explain covariances of items measuring carer wellbeing, presumably because they represented the core meaning of QOL. This is not a problem but rather success in capturing ‘pure’ meaning of QOL by a subset of items. Supplementary Table S1 presents the standardized factor loadings for the final C-DEMQOL bifactor measurement model.

### Scoring C-DEMQOL and norm referencing

Because the response options have a natural order from best QOL to worst, the items can be scored by assigning consecutive integers from 5 to 1. Although the integers arising from the expanded response format cannot be referred back to same category labels, they can be scaled using standard methods for Likert items [12, 19]. For items that satisfied the requirement of homogeneity (measuring one construct in common) in factor analysis, scale scores can be computed by either non-linear methods taking into account the ordinal nature of item responses (i.e. Item Response Theory) or by linear approximation such as summated scales [19]. The latter method is straightforward to apply to C-DEMQOL items, since they satisfy another requirement for summated scales—that ‘each item should have the same possible range of score values’ [12, p. 216].

To calculate the total QOL score, all 30 item scores are summed. To calculate subdomain scores, items indicating each of the five QOL domains are summed. The use of subdomain scores is justified by the PRMSE_TOT_ of each domain (Table 3) being substantially smaller than the respective reliability, confirming that the subdomain scores are better indicators of the domain constructs than the total score.

**Table 3:** Descriptive statistics, Standard Errors of measurement, reliabilities and attribution of variance of the C-DEMQOL total and subdomain sum scores; construct reliabilities of the C-DEMQOL factors

Statistics	(Total) Overall QOL	(S1) Meeting personal needs	(S2) Carer wellbeing	(S3) Carer-patient relationship	(S4) Confidence in the future	(S5) Feeling supported
Minimum	50	6	6	12	6	6
Maximum	138	30	29	30	30	30
Percentiles						
10	70.03	11.00	9.00	17.00	10.94	12.00
20	79.00	13.00	13.00	18.00	13.00	15.48
30	83.00	15.00	14.00	20.00	15.00	18.00
40	88.98	17.00	16.00	21.00	16.00	19.00
50	96.32	18.00	17.00	22.00	18.00	20.00
60	102.25	19.20	19.00	23.00	20.00	21.76
70	106.87	21.00	21.00	24.00	21.00	24.00
80	114.83	23.00	22.00	25.20	23.00	25.00
90	121.03	25.00	25.00	27.00	25.00	27.00
Mean	95.45	18.24	17.27	21.94	17.90	20.05
SD	19.66	5.30	5.41	3.78	5.34	5.41
Standard error of measurement (SE_M_)	3.60	1.14	1.63	1.62	1.65	2.07
Omega (*ω*)	0.97	0.95	0.91	0.82	0.90	0.85
Omega hierarchical (*ω*_h_ or *ω*_hs_)	0.86	0.45	0.00	0.39	0.33	0.52
PRMSE_TOT_	n/a	0.60	0.75	0.47	0.67	0.50
Construct reliability (H)	0.96	0.79	n/a	0.70	0.68	0.78

For the treatment of *missing data,* we recommend estimating the total and the subdomain scores if at least five item responses per subdomain are present.^2^ Scale scores are estimated as the average of the present responses multiplied by the number of items in the scale (30 for the total scale, and six for each subscale). We therefore recommend mean replacement for missing responses when sum scores are computed, to maintain the unit of measurement and compatibility with the normative reference.

Table 3 reports the descriptive statistics of the C-DEMQOL total and subdomain scores in our sample of 300 carers. The scores were distributed symmetrically, with no notable deviations from normality, which suggests that the presented means, SDs and percentiles can be used as an interim normative reference until such time as representative normative data are collected for C-DEMQOL.

### Reliability and standard error of measurement

Table 3 reports reliability estimates for the C-DEMQOL scores. For the total QOL score, omega is *ω* = 0.97, and for the subdomain scores, omegas range from 0.82 to 0.95. For reference and comparability with other measures, coefficients alpha are also reported in Table 4; however, alpha underestimates reliability when factor loadings are not equal [31].

**Table 4:** Correlations between C-DEMQOL sum scores and validation measures

Measures	Alpha	(Total) Overall QOL	(S1) Meeting personal needs	(S2) Carer wellbeing	(S3) Carer-patient relationship	(S4) Confidence in the future	(S5) Feeling supported
*C*-*DEMQOL scales*							
C-DEMQOL Total	0.93						
C-DEMQOL S1	0.93	0.77					
C-DEMQOL S2	0.87	0.87	0.67				
C-DEMQOL S3	0.74	0.70	0.38	0.56			
C-DEMQOL S4	0.87	0.82	0.49	0.70	0.52		
C-DEMQOL S5	0.80	0.73	0.43	0.47	0.45	0.49	
*Carer measures (self*-*report)*							
GHQ-12^(a)^	0.88	− 0.63	− 0.45	**− 0.61**	− 0.41	**− 0.57**	− 0.40
Hospital anxiety and depression scale^(a)^	0.89	− 0.71	− 0.55	**− 0.65**	− 0.43	**− 0.63**	− 0.47
Zarit carer burden inventory^(a)^	0.91	− 0.79	**− 0.65**	**− 0.68**	**− 0.60**	**− 0.69**	− 0.49
WHOQOL physical health	0.83	0.61	0.47	**0.51**	0.42	0.53	0.42
WHOQOL psychological	0.83	0.63	0.46	**0.55**	0.45	0.54	0.47
WHOQOL social relationships	0.69	0.45	0.36	0.32	0.33	0.33	**0.44**
WHOQOL environment	0.74	0.57	**0.47**	0.45	0.34	0.46	**0.48**
SF12 physical	0.85	0.39	0.30	0.30	0.27	0.33	0.30
SF12 mental	0.80	0.70	0.53	**0.65**	0.47	0.61	0.48
Personal Wellbeing scale	0.74	0.63	0.44	**0.56**	0.42	0.54	0.49
BADLS help^(a)^	0.89	− 0.32	**− 0.53**	− 0.23	− 0.10ns	− 0.20	− 0.15
*Person with dementia measures (report by proxy)*							
DEMQOL-proxy	0.91	0.23	0.13	0.18	0.10ns	0.24	0.21
Neuropsychiatric inventory^(a)^	0.79	− 0.45	− 0.30	− 0.47	− 0.46	− 0.32	− 0.26
Clinical dementia rating^(a)^	0.95	− 0.08ns	− 0.13	− 0.17	− 0.07ns	0.05ns	0.03ns
BADLS dependence^(a)^	0.96	− 0.12	− 0.20	− 0.22	− 0.09ns	0.05ns	0.04ns

(a) construct is keyed in the opposite direction to C-DEMQOL (i.e. indicates distress or low QOL). Correlations for such constructs have been reversed when computing the average convergent/discriminant correlation. Hypothesized convergent relationships with C-DEMQOL subscale scores used to compute convergent correlations are bold. The C-DEMQOL total score is not included in calculations, because the subscale scores already account for the total variance. (ns) Correlations are not significant at the 0.05 level, two-tailed. All other correlations are significant

Standard errors of measurement (SE_M_) for each scale are reported in Table 3. The SE_M_ can be used for calculating confidence intervals containing the true score.

### Convergent and discriminant validity

Given the high omega hierarchical *ω*_h_ = 0.86, variability in the QOL total score is mostly due to the general factor. Variability in subdomain scores is due to both the general and the specific factors, which contribute important but more modest portions of variance (*ω*_hs_ range from 0.33 to 0.52). These are important considerations for understanding the extent and the sources of C-DEMQOL’s relationships with external measures.

Table 4 presents the inter-scale correlations for the C-DEMQOL total and subdomain scores, and their correlations with the validation measures. Cronbach’s alpha for all measures are reported in the same table. Relationships between conceptually similar (*convergent*) constructs are shaded. The convergent relationships involve the carer-focused measures only; the patient-focused measures, while they may causally relate to carer QOL, are distinct constructs. A detailed mapping between C-DEMQOL and the validation measures with example items is provided in Supplementary Table S2.

Correlations between C-DEMQOL and carer-focused external scales are positive and substantial. The average convergent correlation is 0.58, and the average discriminant is 0.40. The rather large discriminant correlation is not surprising since all the C-DEMQOL subdomains carry substantial variance due to overall QOL. Thus, the source of common variance with external measures is masked when the sum scores are used—it is unclear whether the relationship is due to general QOL or the specific domains controlling for QOL.

To reveal the source of common variance with external scales, we partitioned the variance in C-DEMQOL scores by using the bifactor model. Since the construct reliability (index *H*) for the C-DEMQOL general and domain-specific factors are very close or exceeding 0.7 (see Table 3), all the factors are reliable enough for the use in latent variable models. Table 5 presents correlations between C-DEMQOL latent factors and the validation measures’ observed scores.^3^ Here, correlations for conceptually similar carer-focused scales are much stronger than the correlations for dissimilar scales, with the average convergent correlation high at 0.50 and the discriminant correlation low at 0.15.

**Table 5:** Correlations between C-DEMQOL latent factors and validation measures

Measures	(G) Overall QOL	(S1) Meeting personal needs	(S2) Carer wellbeing	(S3) Carer-patient relationship	(S4) Confidence in the future	(S5) Feeling supported
*Carer measures (self*-*report)*						
GHQ-12^(a)^	**− 0.66**	0.04ns	–	0.03ns	**− 0.21**	− 0.02ns
Hospital anxiety and depression scale^(a)^	**− 0.70**	− 0.17	–	0.06ns	**− 0.37**	− 0.13ns
Zarit carer burden inventory^(a)^	**− 0.73**	**− 0.45**	–	**− 0.46**	**− 0.47**	− 0.21
WHOQOL physical health	**0.56**	0.16	–	0.15ns	0.26	0.18
WHOQOL psychological	**0.61**	0.07ns	–	0.18	0.22	0.20
WHOQOL social relationships	0.36	0.17	–	0.17	0.09ns	**0.31**
WHOQOL environment	0.50	**0.24**	–	0.08ns	0.17	**0.31**
SF12 physical	0.34	0.11ns	–	0.08ns	0.13ns	0.15
SF12 mental	**0.70**	0.09ns	–	0.06ns	0.27	0.17ns
personal wellbeing scale	**0.60**	0.01ns	–	0.07ns	0.25	0.30
BADLS help^(a)^	− 0.26	**− 0.55**	–	0.12ns	0.01^ns^	0.03^ns^
*Person with dementia measures (report by proxy)*						
DEMQOL-proxy	0.21	− 0.02ns	–	− 0.01ns	0.15	0.12ns
Neuropsychiatric inventory^(a)^	− 0.45	0.02ns	–	− 0.36	0.04ns	0.03ns
Clinical dementia rating^(a)^	− 0.14	− 0.06ns	–	− 0.02ns	0.28	0.13
BADLS dependence^(a)^	− 0.18	− 0.12	–	− 0.01ns	0.33	0.19

(a) construct is keyed in the opposite direction to C-DEMQOL (i.e. indicates distress or low QOL). Correlations for such constructs have been reversed when computing the average convergent/discriminant correlation. Hypothesized convergent relationships with C-DEMQOL factors are bold. (ns) Correlations are not significant at the 0.05 level, two-tailed. All other correlations are significant

Relationships with the patient-focused scales, also given in Tables 4 and 5, provide additional evidence for external validity of C-DEMQOL general and specific domains. Carer-reported problems experienced by the person with dementia have an impact on the carer QOL. In addition, behaviours such as aggression, agitation or irritability shown by the person with dementia (measured by the Neuropsychiatric Inventory) have an impact on the relationships between the carer and the person with dementia.

## Discussion

We have addressed the current need for condition-specific measurement of the quality of life of carers of people with dementia by developing and validating a new instrument using a sequential ‘bottom-up’ approach. The development was grounded in the experiences of family carers from the outset by exploring issues relevant to the Carers’ QOL and analysing them qualitatively. We also piloted and discussed the successive questionnaire versions with the participating carers and the LEAP. We adopted a thorough and careful approach to data collection, involving the total of 447 one-to-one interviews with carers, each lasting up to 2 h, performed by two experienced research workers over a 2-year period. Quantitative analyses involved modern psychometric methods recommended as the gold standard for clinical measurement [27, 28].

The resulting C-DEMQOL demonstrated high reliability and validity in people caring across different situations (co-resident/non-resident; spouse/next generation; rural/urban) and for people with different severities of dementia. The total score is the most robust and reliable (*ω* = 0.97) measure that C-DEMQOL provides and can be used for assessing overall QOL across all domains. If the focus of measurement is on specific domains, subdomain scores can be computed, and our analyses show these enhance information over and above the total score.

The results presented here are encouraging and suggest that C-DEMQOL is likely to be a valuable instrument to use in research. This could include studies investigating the impact on carer QOL of interventions for people with dementia or for carers themselves, and other descriptive and evaluative work that seeks to understand the carer experience.

### Using C-DEMQOL

We estimate that completing C-DEMQOL in an interview will take most respondents less than 15 min, with the average respondent completing in 12 min. The questionnaire is designed to be suitable for self-completion; however, this mode of administration was not tested in the present study. Responses can be scored by hand or computer, and the total QOL score as well as five subscale scores can be presented. The measure and the users’ manual are available at www.bsms.ac.uk/cdemqol.

We designed C-DEMQOL so that it enables the use of summated scores as the most practical option in applied settings. The present paper provides the corresponding coding and scoring instruction, the interim normative reference and standard errors of measurement. However, the use of C-DEMQOL is not limited to sum scores. The bifactor measurement model illustrated in Fig. 1 can be used for model-based measurement taking full account of the ordinal nature of these data (Item Response Theory or IRT), not only for establishing item parameters and computing coefficients omega as we did in this research, but also for scoring. IRT methods can be used to estimate the most likely trait score given the response pattern (by either maximum likelihood or Bayesian methods). These scores have many advantages well described in the literature, including but not limited to establishing confidence intervals around a score based on the Standard Error unique to a particular response pattern [19], which aids interpretation of an individual score, for example its responsiveness to change.

C-DEMQOL can be used on its own or with DEMQOL and DEMQOL-Proxy as a suite of complementary QOL measures that together provide a comprehensive disease-specific profile of QOL for people with dementia and their family carers for use in research and clinical settings. By using instruments that capture these attributes more fully and more accurately, we can build a deeper and broader understanding of QOL in dementia, for both people with dementia and family carers, and how interventions affect them individually and together. Future research could also see the development of C-DEMQOL into a preference based instrument, which can be used for economic evaluation, alongside DEMQOL-U and DEMQOL-Proxy-U [21, 29].

### Limitations and strengths

There are important limitations in the research presented. First, despite targeted sampling to include important subpopulations, not all subgroups of family carers will have been represented. Further work is needed to evaluate the psychometric properties of C-DEMQOL in subgroups such as those caring for young-onset dementia, people with dementia from minority ethnic communities and in different conditions such as Lewy Body dementia or frontotemporal dementia. Second, instruments used in evaluative studies need to be responsive to change. It was not possible to examine C-DEMQOL’s responsiveness within this study. To evaluate responsiveness, the instrument would need to be tested in an intervention designed to improve family carer QOL of known effectiveness. We are in the process of including C-DEMQOL in such studies, but until data are available, the instrument should be considered experimental. Third, we have developed and tested a UK English version of C-DEMQOL. There will be a need for careful cross-cultural adaptation if the instrument is to be used in other territories [15]. Fourth, while the results presented here are highly encouraging, there is a need for replication in independent samples. Replication of the bifactor measurement model is particularly important, because in this study, the final item reduction and CFA were performed on the same dataset, thus potentially optimizing the model fit. Validation against external measures would also benefit from replication; however, our validation results are likely robust, since the correlations with external measures were computed after any optimisation of the questionnaire structure (and this optimisation was blind to the validation data).

These limitations notwithstanding, there are strengths in the approaches taken. We followed best practice in questionnaire development and evaluated the instrument’s psychometric properties extensively based on a well-fitting measurement model, and the results were consistently positive. While requiring further validation, C-DEMQOL represents a step-change compared with the available questionnaires to measure the QOL of family carers of people with dementia.

### Electronic supplementary material

Below is the link to the electronic supplementary material.

Supplementary material 1 (DOCX 30 kb)

## Appendix: sociodemographic and validation measures used in Stages 3 and 4

1. DEMQOL-Proxy: QOL of the person with dementia [32];
2. Neuropsychiatric Inventory (NPI): symptoms in the person with dementia [6];
3. Clinical Dementia Rating (CDR): dementia severity [20];
4. (Modified) Bristol Activities of Daily Living Scale (BADLS): degree of dependence of person with dementia, and amount of help carer provides [4];
5. Client Service Receipt Inventory (CSRI): carer demographics and service use [3];
6. General Health Questionnaire (GHQ-12): carer mental health [13];
7. Hospital Anxiety and Depression Scale (HADS): carer depression and anxiety [40];
8. Zarit Carer Burden Inventory (ZCBI): carer burden [38];
9. World Health Organization Quality of Life Assessment (WHOQOL-BREF) [36];
10. Short Form Health Survey (SF-12v2): carer health-related QOL [35];
11. Personal Wellbeing Scale: carer wellbeing [9].

## Footnotes

1 We use ‘family carer’ to include all informal (unpaid) carers (i.e. family and friends) of a person with dementia. The term ‘carer’ was preferred to ‘caregiver’ and ‘family’ to ‘informal’ by our Lived Experience Advisory Panel.

2 Allowing only one missing response per subdomain may be unnecessarily restrictive when computing the total score. Given the strong saturation of the general factor, the meaning and the scale of overall QOL might not be invalidated by subdomain under-representation. However, in these early days of C-DEMQOL we prioritize content coverage, but suggest for this recommendation to be empirically examined in future research.

3 These were computed by regressing the latent factors in the bifactor model on sum scores of validation measures, one at a time, and standardizing the obtained regression coefficient.
