# Supplementary material for: Measuring the quality of life of family carers of people with dementia: development and validation of C-DEMQOL
Source: Qual Life Res. 2019 Apr 27;28(8):2299–310. doi: 10.1007/s11136-019-02186-w (PMC6620239; doi:10.1007/s11136-019-02186-w)
Supplement: Supplementary file 1 — Supplementary material 1 (DOCX 30 kb) [file 11136_2019_2186_MOESM1_ESM.docx]

**Table S1**. Standardized factor loadings in the C-DEMQOL bifactor measurement model

| Item content | (G) QOL | (S1) Meeting personal needs | (S2)  Carer wellbeing | (S3)  Carer-patient relationship | (S4) Confidence in the future | (S5)  Feeling supported |
| --- | --- | --- | --- | --- | --- | --- |
| 1. Energy used caring | .68 | .49 |  |  |  |  |
| 1. Time used caring | .53 | .64 |  |  |  |  |
| 1. Meeting own needs | .69 | .57 |  |  |  |  |
| 1. Ability to do things I enjoy | .63 | .70 |  |  |  |  |
| 1. Impact on freedom | .63 | .65 |  |  |  |  |
| 1. Needs for activities met | .67 | .58 |  |  |  |  |
| 1. Emotional demands | .86 |  | (.00) |  |  |  |
| 1. Stress of meeting demands | .91 |  | (.00) |  |  |  |
| 1. Emotional problems | .83 |  | (.00) |  |  |  |
| 1. Guilt when getting away | .58 |  | (.00) |  |  |  |
| 1. Upset due to dementia | .68 |  | (.00) |  |  |  |
| 1. Impact of caring on health | .85 |  | (.00) |  |  |  |
| 1. Coping with caring demands | .54 |  |  | .26 |  |  |
| 1. Acceptance of caring role | .56 |  |  | .34 |  |  |
| 1. Change in relationship* | .31 |  |  | .30 |  |  |
| 1. Relationship currently | .45 |  |  | .74 |  |  |
| 1. Appreciated by PWD | .39 |  |  | .63 |  |  |
| 1. Frustration toward PWD | .53 |  |  | .37 |  |  |
| 1. Worry about meeting future needs of PWD | .63 |  |  |  | .51 |  |
| 1. Coping emotionally in future | .61 |  |  |  | .53 |  |
| 1. Worries for PWD in future | .55 |  |  |  | .66 |  |
| 1. Difficulty meeting future needs of PWD | .66 |  |  |  | .44 |  |
| 1. Making important decisions | .66 |  |  |  | .51 |  |
| 1. Future financial impact | .59 |  |  |  | .17 |  |
| 1. Overall needs for support met | .64 |  |  |  |  | .40 |
| 1. Professional support met expectations | .46 |  |  |  |  | .79 |
| 1. Supported by professionals | .47 |  |  |  |  | .70 |
| 1. Supported by family | .45 |  |  |  |  | .34 |
| 1. Supported by friends | .38 |  |  |  |  | .44 |
| 1. Supported by wider networks* | .17 |  |  |  |  | .53 |

NOTE. PWD = Person with dementia. Factor loadings in parentheses (.00) are fixed. *Change in wording for this item is recommended for the future use of C-DEMQOL.

**Table S2.** Map of conceptual concordance of the C-DEMQOL domains and external measures

|  | (S1) Meeting personal needs | (S2) Carer wellbeing | (S3) Carer-patient relationship | (S4) Confidence in the future | (S5) Feeling supported |
| --- | --- | --- | --- | --- | --- |
| *Carer measures (self-report)* | | | | | |
| GHQ-12 * |  | most items |  | 10. Been losing confidence in yourself?  6. Felt you couldn’t overcome your difficulties?  4. Felt capable of making decisions about things? |  |
| Hospital Anxiety and Depression Scale * | 14 I can enjoy a good book, radio or TV programme;  2 I still enjoy the things I used to enjoy | most items |  | 5 Worrying thoughts go through my mind |  |
| Zarit Carer Burden Inventory * | 2 Do you feel that because of the time you spend with (the participant) that you do not have enough time for yourself?  17 Do you feel you have lost control of your life since (the participant)’s illness? | 9 Do you feel strained when you are around (the participant)?  10 Do you feel your health has suffered because of your involvement with (the participant)? | 5 Do you feel angry when you are around (the participant)?  6 Do you feel that (the participant) currently affects your relationship with other family members or friends in a negative way?  21 Do you feel you could do a better job in caring for (the participant)? | 7 Are you afraid what the future holds for (the participant)?  15 Do you feel that you do not have enough money to care for (the participant), in addition to the rest of the expenses?  16 Do you feel that you will be unable to take care of (the participant) much longer?  19 Do you feel uncertain about what to do about (the participant)? |  |
| WHOQOL Physical Health |  | 16 How satisfied are you with your sleep? |  |  |  |
| WHOQOL Psychological |  | 5 How much do you enjoy life? 26 How often do you have negative feelings, such as blue mood, despair, anxiety, depression? |  |  |  |
| WHOQOL Social Relationships |  |  |  |  | 20 How satisfied are you with your personal relationships? 22 How satisfied are you with the support you get from your friends? |
| WHOQOL Environment | 14 To what extent do you have the opportunity for leisure activities? |  |  |  | 13 How available to you is the information that you need in your day-to-day life?  24 How satisfied are you with your access to health services? |
| SF12 Physical |  | 7. has your physical health or emotional problems interfered with… |  |  |  |
| SF12 Mental |  | 4 problems with your work or other regular daily activities as a result of any emotional problems (such as feeling depressed or anxious)? 6a Have you felt calm and peaceful? 6c Have you felt downhearted and low? 7. has your physical health or emotional problems interfered with ... |  |  |  |
| Personal Wellbeing Scale |  | 3. Overall, how happy did you feel yesterday? 4. overall, how anxious did you feel yesterday? |  |  |  |
| BADLS Help* | all items |  |  |  |  |
| *Person with Dementia measures (report by proxy)* | | | | | |
| DEMQOL-Proxy |  | PWD poor QOL can impact carer wellbeing; can cause carer to "find changes upsetting" |  |  |  |
| Neuropsychiatric Inventory * |  | PWD problems with sleep or depression can impact carer wellbeing; other problems can cause carer to "find changes upsetting" | PWD aggression, agitation, irritability, depression can impact relationship with carer |  |  |
| Clinical Dementia Rating* |  | Severe dementia symptoms can cause carer to "find changes upsetting" |  |  |  |
| BADLS Dependence* | PWD dependence can impact carer's personal freedom (if the carer is the person who assists - this is unknown from the Dependence scale alone) | PWD dependence can impact carer wellbeing (CDEMQOL "stress of meeting demands"; "find changes upsetting"; "guilt when getting away") |  |  |  |

NOTE. * External construct is keyed in the opposite direction to C-DEMQOL (i.e. indicates distress or low QOL). Correlations for such constructs must be reversed when computing the average convergent/discriminant correlation.

Shading: Grey = May be causally related (applies to patient-centred measures only); Yellow = potential overlap in definition; Orange = substantial overlap in definition OR essentially the same construct.
